# Polymer Physics Models Reveal Structural Folding Features of Single-Molecule Gene Chromatin Conformations

**DOI:** 10.3390/ijms251810215

**Published:** 2024-09-23

**Authors:** Mattia Conte, Alex Abraham, Andrea Esposito, Liyan Yang, Johan H. Gibcus, Krishna M. Parsi, Francesca Vercellone, Andrea Fontana, Florinda Di Pierno, Job Dekker, Mario Nicodemi

**Affiliations:** 1 Dipartimento di Fisica, Università di Napoli Federico II, and INFN Napoli, Complesso Universitario di Monte Sant’Angelo, 80126 Naples, Italy; 2 Department of Systems Biology, University of Massachusetts Chan Medical School, Worcester, MA 01605, USA; 3 Diabetes Center of Excellence and Program in Molecular Medicine, University of Massachusetts Chan Medical School, Worcester, MA 01655, USA; 4 DIETI, Università di Napoli Federico II, Via Claudio 21, 80125 Naples, Italy; 5 INFN Napoli, Complesso Universitario di Monte Sant’Angelo, 80126 Naples, Italy; 6 Howard Hughes Medical Institute, Chevy Chase, MD 20815, USA

**Keywords:** chromatin folding, polymer physics, phase separation, gene regulation, epigenetics

## Abstract

Here, we employ polymer physics models of chromatin to investigate the 3D folding of a 2 Mb wide genomic region encompassing the human *LTN1* gene, a crucial DNA locus involved in key cellular functions. Through extensive Molecular Dynamics simulations, we reconstruct in silico the ensemble of single-molecule *LTN1* 3D structures, which we benchmark against recent in situ Hi-C 2.0 data. The model-derived single molecules are then used to predict structural folding features at the single-cell level, providing testable predictions for super-resolution microscopy experiments.

## 1. Introduction

Recent DNA technologies, such as Hi-C [1,2], GAM [3,4], and SPRITE [5,6], have shown that mammalian chromosomes have complex, non-random 3D architectures within the cell nucleus, encompassing multiple folding structures across genomic scales [7,8,9,10,11,12,13]. Such an organization includes, for example, DNA loops [14], Topologically Associating Domains (TADs) [15,16], metaTADs [17], and larger architectural features, such as A/B compartments [1], lamina-associated interactions [18], and nuclear chromosome territories [19]. These chromatin structures play critical roles in gene regulation, as distal DNA regulatory elements (e.g., enhancers) have been reported to establish specific physical contacts with their target genes, particularly within TADs, to orchestrate cell transcriptional programs [20,21,22,23,24,25,26,27,28,29,30,31]. Misfolding of chromatin 3D structure, which can lead to abnormal gene–enhancer interactions, has been indeed implicated in genetic disease [32,33,34,35,36,37,38,39,40,41,42,43].

Moreover, advancements in super-resolution microscopy techniques have enabled the direct visualization of chromatin conformations at nanometer scales within individual cell nuclei, providing quantitative insights into single-cell genome structures [44]. These methods have revealed, for instance, that TAD-like domains exhibit spatially segregated globular conformations in single cells, albeit with significant cell-to-cell variability, highlighting crucial constraints on chromatin folding at the level of single DNA molecules [45,46,47,48,49,50,51,52,53,54,55,56,57].

On the other hand, theoretical models of chromatin have been instrumental in bolstering experimental technologies to understand the 3D organization of the genome [58]. These models, including polymer physics-based and computational approaches [59,60,61,62,63,64,65,66,67,68,69,70,71,72,73,74,75,76,77,78,79,80,81,82,83,84,85,86,87,88,89,90,91,92,93,94,95,96,97,98,99,100,101,102,103,104,105,106,107,108,109,110,111,112], have been used, e.g., to dissect the fundamental molecular mechanisms shaping DNA contact formation and to derive quantitatively testable predictions, particularly in disease contexts [113,114,115,116,117,118,119,120,121,122,123,124].

In this work, we focus on a reference polymer physics approach, the Strings and Binders (SBS) model of chromatin [86,87], which has been extensively validated against independent experimental datasets, such as bulk Hi-C, GAM, and single-cell microscopy data [125,126,127,128]. In the SBS picture, physical contacts between distal DNA sites (such as genes and enhancers) are established by diffusing cognate binding factors, which can bridge DNA binding sites through thermodynamic mechanisms of phase transitions [61,127]. Here, we apply the SBS model to investigate the single-molecule folding of a 2 Mb wide genomic locus (Chr21:28–30 Mb, hg38) encompassing the *LTN1* gene in human WTC-11 cells (a human-induced pluripotent stem cell line), a crucial locus involved in diverse cellular functions ranging, e.g., from embryonic development and targeting of misfolded proteins to the onset of neurodegenerative diseases [129,130,131,132,133,134,135,136].

By performing massive Molecular Dynamics (MD) simulations of the model, we derive an ensemble of in silico 3D conformation of the gene locus that we validate against recent in situ Hi-C 2.0 data generated via a highly optimized Hi-C protocol [137] available from the 4DN Data Portal (dataset reference number 4DNESJ7S5NDJ) [138,139]. Next, by leveraging the model single-polymer conformations, we conduct structural analyses at the single-molecule level, including spatial distance matrices, assessment of cell-to-cell folding variability, and 3D shape descriptors, producing non-trivial predictions that can be tested by real single-cell microscopy (e.g., super-resolution multiplexed FISH) experiments.

Overall, the model provides a validated, quantitative blueprint for assessing the spatial organization of key human genomic loci at the single-molecule level, complementing empirical investigations in the understanding of chromatin structures in single cells.

## 2. Methods

### 2.1. The Strings and Binders (SBS) Polymer Model

**Figure 1:**
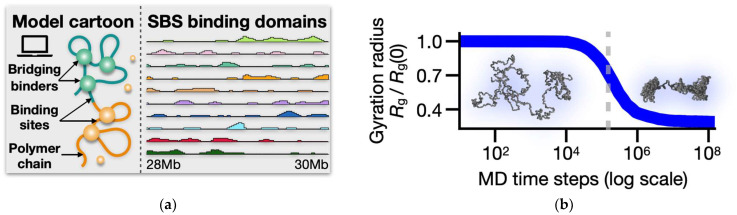
(**a**) In the SBS model, a chromatin region is represented by a self-avoiding polymer chain along which specific binding sites are arranged for diffusing cognate molecular binders. By bridging cognate sites on the chain, the binders drive the folding of the polymer-forming microphase-separated globular structures. The SBS binding domains of the studies *LTN1* locus (Chr21:28–30 Mb) in human WTC-11 cells are shown along with a schematic cartoon of the polymer model. (**b**) The polymer gyration radius *R*_g_ is shown here as a function of the MD time iteration steps (y-axis normalized by the *R*_g_ value at *t* = 0). The function exhibits a sharp drop around 10^5^ time steps, signaling the collapse of the chain from an initial coil (i.e., randomly folded) to an equilibrium globule conformation [140]. A representative coil and phase-separated globule 3D structures are shown, respectively, below and above the phase transition point (gray shaded line in the figure).

The Strings and Binders (SBS) polymer model envisages a theoretical framework for understanding the 3D organization of chromatin where molecular interactions between distant genomic regions are driven by diffusing agents, such as Transcription Factors (TFs) or coactivators, that diffuse in the nuclear environment [86,87]. In the model, a chromosomal segment is represented as a coarse-grained, self-avoiding walk (SAW) polymer chain with specific binding sites for molecular bridging binders (a schematic model cartoon is shown in Figure 1a).

In the simplest case of an SBS homopolymer chain, where the binding sites are all equal, the model has a phase transition from an extended coil (randomly folded) to a compact globular state [140] as soon as the binder molar concentration or its attractive interaction strength exceeds critical thresholds (see also below). For weak biochemical affinities (i.e., units of K_B_T) and for genomic resolutions approaching the sub-megabase scale, these thresholds are in the range of a few micromole/l [61,127], consistent with typical TF concentrations observed in vitro [141].

To dissect the folding dynamics of real chromatin regions characterized by complex genomic contacts, the SBS model can be extended to include multiple types of binding sites, each associated with a specific type of cognate molecular binder [61,142]. Such heteropolymer configurations induce micro-phase separations of the polymer chain, leading to the formation of distinct globular domains enriched with specific binding motifs [127]. The features of these binding sites (i.e., their genomic location and relative abundance) are determined through a previously published machine learning approach, named PRISMR [117], which employs standard Monte Carlo-based optimization procedures to infer the optimal (i.e., minimal) SBS model of specific chromatin regions based solely on input bulk (e.g., Hi-C) contact data, with no additional fitting parameters. In our studied *LTN1* locus in human WTC-11 cells (Chr21: 28–30 Mb), PRISMR returns a polymer of 800 beads with ten distinct types of binding sites, visually represented by different colors (Figure 1a). Interestingly, these domains have an overlapping, combinatorial organization along the chain, which has been shown to be crucial in explaining chromatin contacts with high genome-wide accuracy [143].

### 2.2. Molecular Dynamics (MD) Simulations

To derive a statistical ensemble of single-molecule model 3D structures of the *LTN1* locus, we performed extensive Molecular Dynamics (MD) simulations within the freely available LAMMPS software (version 30 July 2016) [144], optimized for parallel computation [145,146]. In the model MD implementation, the polymer is a standard coarse-grained bead-spring chain [147], and the binders are simple spherical particles. The motion of polymer beads and binders is determined by a Langevin equation with standard parameters (friction coefficient *ζ =* 0.5 and temperature *T* = 1, dimensionless units), numerically integrated via the Velocity–Verlet algorithm [148]. For simplicity, in our simulations, we set the diameter of both polymer beads and binders to *σ* = 1, using it as the unit length of the model; similarly, we set the mass of beads and binders to be equal, taking it as the reference mass unit *m* = 1 [147].

The physical interaction potentials are set as in classical polymer simulation studies [147]. Excluded volume interactions between consecutive beads are modeled using a truncated, purely repulsive Lennard–Jones (LJ) potential, adjacent polymer beads are connected by FENE bonds with standard parameters (maximum length 1.6*σ* and spring constant 30K_B_T/*σ*^2^), and attractive interactions between beads and cognate binders are described by a short-range, truncated LJ (cut-off distance 1.5*σ*).

The initial states of the MD simulations are open SAW configurations, located in a cubic simulation box of size 50*σ* with periodic boundary conditions to control finite size effects [149]. Binders are randomly injected into the simulation box, and then the system of beads and binders is equilibrated for 10^8^ MD time iteration steps. To monitor the system folding dynamics, we recorded the time track of the polymer gyration radius, *R*_g_. This function has a sharp drop at a characteristic time scale and then plateaus, marking the phase transition of the polymer from an initial free SAW chain to an equilibrium phase-separated globular conformation (Figure 1b). To give a sense of the chain compaction, the linear size of the polymer decreases by around 70% in the transition [140], resulting in an average *R*_g_ in the phase-separated state equal to 6.4*σ*.

**Figure 2:**
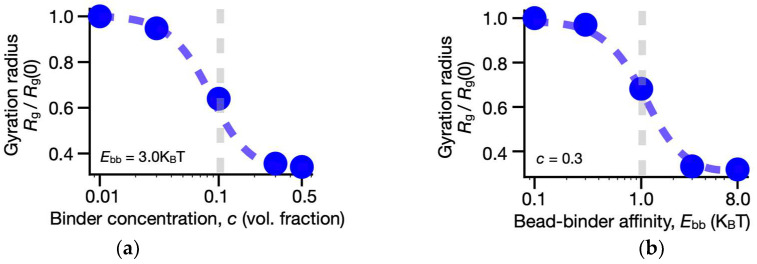
The gyration radius of the polymer chain is shown here as a function of (**a**) the binder concentration, *c* (expressed in volume fraction), and (**b**) the bead-binder affinity (*E*_bb_, in K_B_T units). For *c* ≃ 0 (i.e., no binders), or analogously for *E*_bb_ ≃ 0 (i.e., no bead-binder attractions), the polymer is a randomly folded chain (in the SAW universality class), as only random and fleeting contacts are established in the absence of binders [140]; as soon as the number of binders (or their energy affinity) grows above a characteristic threshold, the polymer collapses into an equilibrium globular state where specific contacts are established based on the underlying distribution of the inferred binding sites (see above). The characteristic threshold concentrations and affinities depend on model details: for the considered model parameters, they fall, respectively, around 0.1 and 1.0 K_B_T.

To test model robustness, we sampled a broad range of bead-binder affinity values in the weak biochemical energy scale, from 0 to 8 K_B_T, and explored up to three orders of magnitude in binder concentration as detailed in previous studies [61,127,150]. As explained above, the number of binders (or their energy affinities) serves as a key control parameter in the model folding, sharply driving its switch-like conformational change from the initial coil to the equilibrium phase-separated globular state, consistent with classic results of polymer phase transitions (Figure 2) [140]. For each set of parameters, we performed up to 3 × 10^2^ independent runs to ensure statistical strength.

The MD length scale (i.e., the bead diameter *σ*) can be mapped into physical units using the relation [87] *σ* = (*s/G*)*D*^1/3^, where *s* is the genomic content per bead (*s* = *L/N*, where *L* = 2 Mb is the genomic length of the locus and *N* = 800 the number of beads), *G* is the genome length (6 Gb), and *D* is the cell nucleus diameter (taken to be 10 μm as an order of magnitude). These approximations yield *σ* = 74.7 nm, which is in line with previous polymer physics studies [77,83]. As an additional check, single-cell microscopy data of the same *LTN1* genomic region in human IMR90 cells reported an average *R*_g_ of the locus equal to 464 nm [45]. This value, matched with that found in our simulations (6.4*σ*), returns *σ* = 72.5 nm, which is remarkably close to our previous calculation. In the following, to set a reference value for *σ*, we take the average of those two independent estimates, i.e., *σ* = 73.6 nm.

Finally, the simulation time, *τ*, can be converted into physical time via the formula *τ =* 6π*ησ*^3^*/(K_B_T)*, where *η* is the nucleoplasm viscosity. Given typical viscosity values in the range of a few fractions of poise [63,151], the model time scale is on the order of milliseconds, which is consistent with classic chromatin simulations [63].

## 3. Results

### 3.1. Folding of the SBS Model of the Human LTN1 Gene Locus

To comprehensively investigate the folding properties of our model at the ensemble population level, we measured the average pairwise contact matrix in its phase-separated state. This was achieved by computing the mean of the model single-molecule contact maps, i.e., symmetric square matrices where each entry, *A*_ij_, is either 1 or 0, depending on whether the polymer sites *i* and *j* are in contact. A contact event is considered to occur if the spatial distance between the sites is below a typical distance threshold [63,117]. For our study, we explored thresholds within the range from 2 up to 5*σ*, corresponding to a spatial distance range of about 150–350 nm (see above), and they all provided analogous results. This range is consistent with established reference contact threshold values from microscopy studies [51].

**Figure 3:**
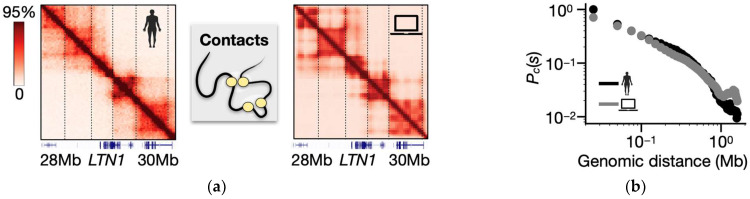
(**a**) In situ Hi-C 2.0 contact data of the studied 2 Mb wide *LTN1* locus in WTC-11 cells (left) are consistently captured by the SBS polymer model (right). The high Pearson and genomic distance corrected correlation values (respectively, r = 0.90 and r’ = 0.59) indicate that the model accurately captures the overall structure of *LTN1* pairwise interactions. (**b**) Comparison between the model and Hi-C contact probabilities at the *LTN1* locus in WTC-11 cells. Overall, the model consistently recapitulates the experimental profile across genomic scales (r = 0.98).

To benchmark and validate the model output, we used publicly available in situ Hi-C 2.0 data [137] of the *LTN1* locus in human WTC-11 cells from the 4DN Data Portal (dataset reference number 4DNESJ7S5NDJ) [138]. The experimental Hi-C contact map, which is binned at a 25 kb resolution to match the model coarse-graining level, exhibits specific and non-random patterns of contacts, including the presence of TADs and sub-TAD domains, inter-TAD interactions, and long-range (>500 kb) looping contacts, particularly around the *LTN1* gene (Figure 3a, left panel).

Remarkably, the contact map generated by the model captures these observed experimental features, successfully recapitulating the overall structure of pairwise interactions (Figure 3a, right). This agreement is quantitatively supported by the high Pearson correlation coefficient between the model and Hi-C contact matrices, r = 0.90. As an additional measure of similarity, we computed the genomic distance corrected Pearson correlation coefficient, r’, which averages out trivial genomic proximity effects [117]. Despite the minimal ingredients of the model, we find r′ = 0.59, which is a comparatively high correlation value considering that a randomly folded control chain would produce an r’ close to zero [152].

To further validate model robustness, we also calculated the Spearman correlation coefficient, r_s_, between the model and experimental contact maps. Our analysis yields a high r_s_ value of 0.76, indicating a strong statistical similarity [97,153].

Finally, to check the performance of the model at different genomic length scales, we performed a more local analysis by computing the contact probability, *P_c_*(*s*), of the model and Hi-C 2.0 data. Our analysis confirms that the model closely aligns with experimental data across three orders of magnitude of genomic separations (Figure 3b; Pearson corr. between the curves r = 0.98).

To summarize, the ensemble of model conformations of the *LTN1* locus aligns well with Hi-C experimental data, demonstrating that our model quantitatively captures in silico the structural features of the locus folding at the population-average level.

### 3.2. Structural Heterogeneity of LTN1 Single-Molecule Conformations in the Model

**Figure 4:**
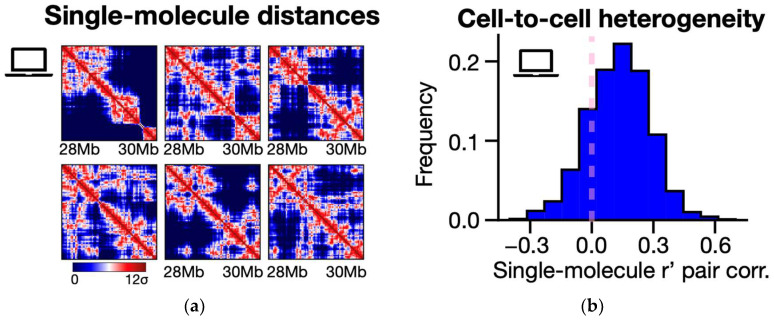
(**a**) Representative examples of model-predicted phase-separated single-molecule distance matrices of the *LTN1* locus. The interaction patterns broadly differ across the ensemble of polymer configurations, as the system can fold in a variety of 3D architectures [127]. (**b**) The structural heterogeneity of individual polymer structures is measured in the model by computing the r’ correlation between pairs of single-molecule distance matrices. The resulting distribution is broad (variance = 0.15) and has a non-zero average value (mean = 0.13), indicating that chromatin structures are highly variable from cell to cell yet have a residual degree of structural correlation.

Next, we aimed to study the folding dynamics of the locus as predicted by our simulations at the single-molecule level. By leveraging the 3D coordinates generated through the SBS model, we computed spatial distance maps for each phase-separated polymer conformation. These maps are symmetric square matrices of pairwise Euclidean distances between polymer sites across the locus. Our analysis reveals distinct single-molecule distance patterns, featuring TAD-like domains occurring at different genomic positions and long-range loop contacts spanning across TAD boundaries (Figure 4a). Consistent with previous studies [45,53,154,155], this variability underscores the highly dynamic nature of the 3D structures of single chromatin conformations. In the SBS model, such a structural heterogeneity arises, beyond stochastic thermal fluctuations, from the inherent folding degeneracy due to the specific, overlapping distribution of its binding sites (see, e.g., Figure 1a) [127].

To quantify the extent of structural variability among model single molecules, we measured the r’ pairwise correlations between their distance matrices. In the absence of a correlation between matrices, we would expect an r’ distribution centered around zero. Conversely, perfectly correlated matrices would yield an r’ distribution peaked at 1. Interestingly, our calculations return an unimodal distribution with a mean value of 0.13 and a variance of 0.15 (Figure 4b), which suggests a substantial heterogeneity of polymer 3D structures that, though, retain a residual degree of structural correlation (as indicated by the non-zero r’ average value). These results are in line with recent microscopy studies that reported a strong cell-to-cell variability of individual chromatin conformations at the sub-megabase scale [45,46,52], revealing, nevertheless, sub-clusters of structures with correlated behaviors [51].

To summarize, the model highlights a broad distribution of single-molecule 3D structures at the human *LTN1* locus, providing quantitative predictions (e.g., single-molecule spatial distances and all-against-all pair correlations), which can be directly tested in real biological contexts by independent single-cell microscopy approaches, including, for instance, super-resolution multiplexed FISH or 3D-SIM-based techniques [156,157,158]. Such experimental validation would not only assess the predictive power of the model but also deepen our understanding of the intrinsic structural diversity and dynamic behavior of genomic loci at the single-molecule level.

### 3.3. Shape and Size of 3D Model Single Molecules

To further characterize the *LTN1* single-molecule structures predicted by our chromatin model, we calculated their shape and volume. Specifically, for each model conformation in the phase-separated state, we computed the inertia tensor, defined as $I_{\beta \gamma }=\sum_{\alpha =1}^{N}m_{\alpha }\left(r_{\alpha }^{2}\delta_{\beta \gamma }-r_{\alpha \beta }r_{\alpha \gamma }\right)$, where *N* is the number of monomers along the polymer chain, *m_α_ is* the mass of the *α*-th monomer, *r_αβ_* is the *β*-th spatial coordinate, and *β,γ* are the spatial component indexes equal to {0,1,2}. The eigenvalues of this tensor, corresponding to the system principal moments of inertias *I_a_*, *I_b,_* and *I_c_*, can be related through standard textbook formulas to the semi-axes (*a*, *b*, *c*) of a triaxial ellipsoid enclosing the volume contour of a given conformation as follows: $a^{2}=\frac{5}{2N}\left(I_{b}-I_{a}+I_{c}\right),b^{2}=\frac{5}{2N}\left(I_{a}-I_{b}+I_{c}\right)$, and $c^{2}=\frac{5}{2N}(I_{a}-I_{c}+I_{b})$.

**Figure 5:**
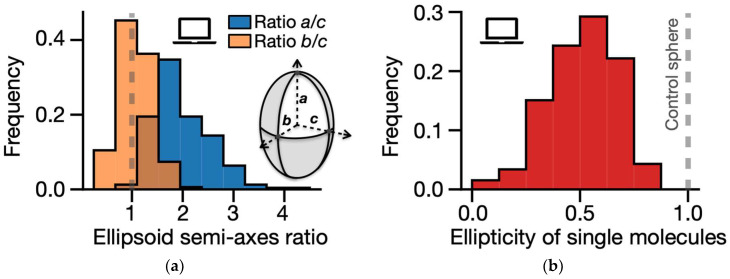
(**a**) Distribution of ellipsoid semi-axis ratios, *a*/*c* (histogram in blue) and *b*/*c* (in orange), computed from the inertia tensor of single-molecule polymer structures. The black dashed line in the figure represents the expected value in the case of perfectly spherical conformations (*a* = *b* = *c*). Interestingly, model-predicted 3D structures of the *LTN1* locus appear prolate, as we find *a* > *b* ≈ *c*. The two distributions, *a*/*c* and *b*/*c,* are statistically different from each other (two-sided Mann–Whitney *p*-value < 0.001). (**b**) The ellipticity of model single molecules, calculated from the eigenvalues of their gyration tensor [159], exhibits a broad distribution (variance = 0.16) with an average value of 0.51, indicating a significant structural variability and a tendency towards a prolate shape.

In the case of a perfectly spherical conformation, the three eigenvalues are equal, and that implies *a* = *b* = *c*. However, in the case of the *LTN1* locus, our model predicts substantial deviations of individual chromatin configurations from a spherical topology, as they are found to have a prolate shape with *a* > *b* ≈ *c* (Figure 5a). Indeed, while the distribution of the ratios *b*/*c* has an average value of 1.0 (orange histogram in the figure), the ratios *a*/*c* are centered around 2.0 (blue histogram); moreover, statistical analysis indicates that these two distributions are significantly distinguishable from each other (two-sided Mann–Whitney *p*-value < 0.001).

Using the inertia tensor, we also computed the volume *V* of single-molecule structures via the formula *V* = 4/3π*abc*, which returns an average volume estimation of the entire locus equal to 1500*σ*^3^. To give a sense of the physical length scales, by taking *σ* = 73.6 nm (see above), the model predicts an average locus volume of approximately 0.59 μm³. We also considered an alternative volume calculation using the formula *V* = 4/3π*R*_g_^3^, which returned a mean *LTN1* volume equal to 1300*σ*^3^ (i.e., 0.52 μm³), comparable to our previous estimate.

Overall, these findings are in line with analogous volume measurements of Mb-wide chromatin loci, which typically fall within the range of fractions of a few μm^3^, as measured, e.g., by super-resolution microscopy and DNAseqFISH+ experiments across different species and cell types [45,51,54].

As an additional test on the shape of model-predicted conformations, we computed the polymer gyration tensor, defined as [159] $T_{\beta \gamma }=\frac{1}{N}\sum_{\alpha =1}^{N}m_{\alpha }\left(r_{\alpha \beta }-{\bar{r}}_{\beta }\right)\left(r_{\alpha \gamma }-{\bar{r}}_{\gamma }\right)$, where *r_αβ_* (*r_αγ_*) is the *β*-th (*γ*-th) component of the position vector of the *α*-th monomer and ${\bar{r}}_{\beta }$ (${\bar{r}}_{\gamma }$) is the component of the mass center of the polymer chain along the *β*-th (*γ*-th) direction. By diagonalizing the tensor, we derived its three eigenvalues, λ_1_ ≥ λ_2_ ≥ λ_3_, from which we calculated the ellipticity, ε, of the model single molecules, defined as ε = 2λ_3_/(λ_1_ + λ_2_). For spherical configurations, ε = 1, as the three eigenvalues are expected to be equal [159]. Consistent with the observed high structural variability of the *LTN1* polymer conformations, we find that the distribution of single-molecule ellipticity values is broad (variance = 0.16), yet it has an average of 0.51, well below that of a spherical control (Figure 5b), highlighting the prolate nature of the single-molecule structures predicted by the model. Targeted microscopy experiments, specifically designed at the *LTN1* region in WTC-11 cells, would provide a direct validation of those structural predictions.

Taken together, these results indicate that the *LTN1* locus in human WTC-11 cells significantly deviates from a simple spherical geometry, predominantly displaying a prolate shape with substantial single-molecule structural variability, as predicted by our chromatin model and tested through multiple, independent shape and volume metrics.

## 4. Discussion

In this work, we combined polymer physics models of chromatin and computer simulations to comprehensively analyze the folding properties of the *LTN1* gene locus in a human-induced pluripotent stem cell line. By computing the average pairwise contact matrix of the model, we demonstrated that its predicted patterns are consistent with available in situ Hi-C 2.0 experiments [137,138], capturing with high accuracy the complex architecture of TADs, sub-TADs, and long-range looping interactions observed at the gene locus.

Beyond population averages, our model reveals significant structural heterogeneity among individual chromatin 3D structures. This variability is evidenced, for instance, by distinct folding patterns in single-molecule spatial distance matrices and by the broad distribution of their pairwise correlation values. Our results suggest that while the *LTN1* locus exhibits a substantial range of conformations, it also maintains a degree of structural correlation across the ensemble of single molecules, which is consistent with observations from recent microscopy studies [45,51].

Furthermore, our calculations of the inertia and gyration tensors provide quantitative insights into the shape and volume of the locus conformations. The model predicts a prolate shape of single molecules, with an average volume estimate in line with super-resolution imaging data [45,54]. It also provides additional testable predictions, such as the distribution of single-molecule ellipticity values, which can be directly validated by advanced microscopy techniques.

Albeit simplified, the model is robust as it is dictated by thermodynamics, and phase transitions and complex emergent behaviors are common to biological and soft-matter physics systems [160,161,162,163,164,165,166,167,168,169,170,171,172,173,174,175,176,177,178].

These findings have significant implications beyond structural characterization. Understanding the variability and dynamics of genomic loci at the single-molecule level is crucial for elucidating the principles of gene regulation, chromatin organization, and cellular function. The observed heterogeneity in 3D structures, indeed, could influence gene expression patterns, regulatory element interactions, and overall genomic stability.

The ability to predict and verify the structural configurations of genomic loci through principled computational models enhances our capacity to interpret how physical changes in individual chromatin structures can lead to functional outcomes, such as differential gene expression, cellular differentiation, and genetic-disease-associated phenotypes. For instance, the model can be used to detect ectopic gene–enhancer interactions, resulting, e.g., from genomic structural variants (SVs) at transcribing loci, which can contribute to developmental disease phenotypes [117,179,180]. These structural predictions can be experimentally validated through independent Hi-C and single-cell microscopy studies using, for example, patient-derived samples, thus offering molecular insights into disease mechanisms that could guide the development of targeted therapies.

This provides a robust basis for experimental validation at the single-cell level and a deeper understanding of the dynamic and heterogeneous nature of chromatin 3D architectures.

## Data Availability

The data that support the findings of this study are available from the corresponding author upon reasonable request. Hi-C 2.0 data used in this study are available at the 4DN Data Portal [138] (dataset reference number 4DNESJ7S5NDJ).
